# Investigating surface area and recovery efficiency of healthcare-associated pathogens to optimize composite environmental sampling

**DOI:** 10.1371/journal.pone.0310283

**Published:** 2024-11-08

**Authors:** Monica Y. Chan-Riley, Jonathan R. Edwards, Judith Noble-Wang, Laura Rose

**Affiliations:** 1 Department of Energy, Oak Ridge institute for Science and Education (ORISE), Oakridge, Tennessee, United States of America; 2 Centers for Disease Control and Prevention, National Center for Emerging and Zoonotic Infectious Diseases, Division of Healthcare Quality Promotion, Clinical and Environmental Microbiology Branch, Atlanta, Georgia, United States of America; AIIMS: All India Institute of Medical Sciences, INDIA

## Abstract

Hospital surfaces are known to contribute to the spread of healthcare-associated antimicrobial pathogens. Environmental sampling can help locate reservoirs and determine intervention strategies, although sampling and detection can be labor intensive. Composite approaches may help reduce time and costs associated with sampling and detection. We investigated optimum surface areas for sampling antimicrobial-resistant organisms (AROs) with a single side of cellulose sponge, created theoretical composites (TC) by adding recovery results from multiple optimum areas, then compared the TC to the standard Centers for Disease Control and Prevention sampling method (one sponge using all sides, whole tool; (WT)). Five AROs were evaluated: carbapenemase-producing KPC+ *Klebsiella pneumoniae* (KPC), *Acinetobacter baumannii* (AB), methicillin-resistant *Staphylococcus aureus* (MRSA), vancomycin-resistant *Enterococcus faecalis* (VRE) *and Clostridioides difficile* spores (CD). Steel coupons comprising four surface areas (323; 645; 1,290 and 2,258 cm^2^) were inoculated, dried, and sampled with one sampling pass using the larger side (face) or the smaller side (edge) of a pre-moistened cellulose sponge tool. Based on the optimum areas determined for each organism, composite areas were 1,290 cm^2^ for MRSA and VRE, 1,936 cm^2^ for AB, 2,580 cm^2^ for CD spores and 3,870 cm^2^ for KPC. Total colony forming units (CFU) recovered using a composite approach was greater or comparable than using multiple WT samplings (over the same area as the composite) for MRSA, VRE and AB (130%; 144% and 95%) yet less than if using multiple WT samplings for KP and CD (47% and 66%). We propose a conservative composite sampling strategy if the target organism is unknown; 323 cm^2^ sampling area for each of the four sides of the sponge, (1290 cm^2^ total). The conservative composite sampling strategy improved the recovery of KP (from 47% to 85% of multiple WT samplings), while MRSA, VRE, AB and CD (131%; 144%; 97% and 66%) remained within 5% to that of the optimum area TC.

## Introduction

The Centers for Disease Control and Prevention (CDC) estimates that one in 31 hospital inpatients acquire a healthcare-associated infection (HAI) including antimicrobial-resistant organisms (AROs) [1, 2] and one in nine of these infections is fatal [3].

Many studies have noted that transmission events can be attributed to the healthcare environment as a reservoir, such as high-touch surfaces, medical equipment and water systems [4]. Pinpointing and disinfecting reservoirs within this environment is crucial, as some microorganisms can persist for hours, days, or even months [5–7], posing a risk of transfer to patients either directly or indirectly through healthcare workers and visitors [4, 7]. Although surface sampling is common and sometimes mandated in the food and pharmaceutical industries to monitor cleanliness and quality of production sites, there are no standards for sampling in healthcare settings [8, 9]. Environmental surface sampling in healthcare settings is commonly used for epidemiologic investigations and infection prevention studies [10].

Composite sampling is commonly employed in other types of environmental sampling, especially when aiming to estimate population central tendency (mean and median) or total bioburden by enlarging the sampling area size, thereby expanding the representative population of a single sample that may have previously required more than one sample to be taken [8, 9, 11, 12]. Composite sampling provides flexibility for investigators, offering an alternative sampling consideration that conserves resources, time, and human power. This reduction in samples expedites downstream processing, which is crucial for achieving a quicker resolution in investigations—particularly vital in healthcare settings where timely responses are essential for effective outbreak management.

Composite surface sampling, where all sides of a single sponge sampling tool are used to sample different areas (each side sampling a new surface), was successfully employed in a previous healthcare facility bioburden study. However only a limited evaluation of sampling areas was conducted prior to the study [2]. A previous study of swab sampling revealed that increasing surface areas sampled with the swab resulted in reduced sampling efficiency [13].

In this work we conducted extensive evaluations to determine the optimum surface areas to sample five healthcare-associated AROs and then applied the optimum surface areas for a composite sampling strategy. We then compared the composite sampling to the CDC standard whole tool sampling method. If the target organism for an investigation is not once evaluated in this study, we propose a conservative composite sampling approach.

## Materials and methods

### Study design and sampling

**Fig 1:**
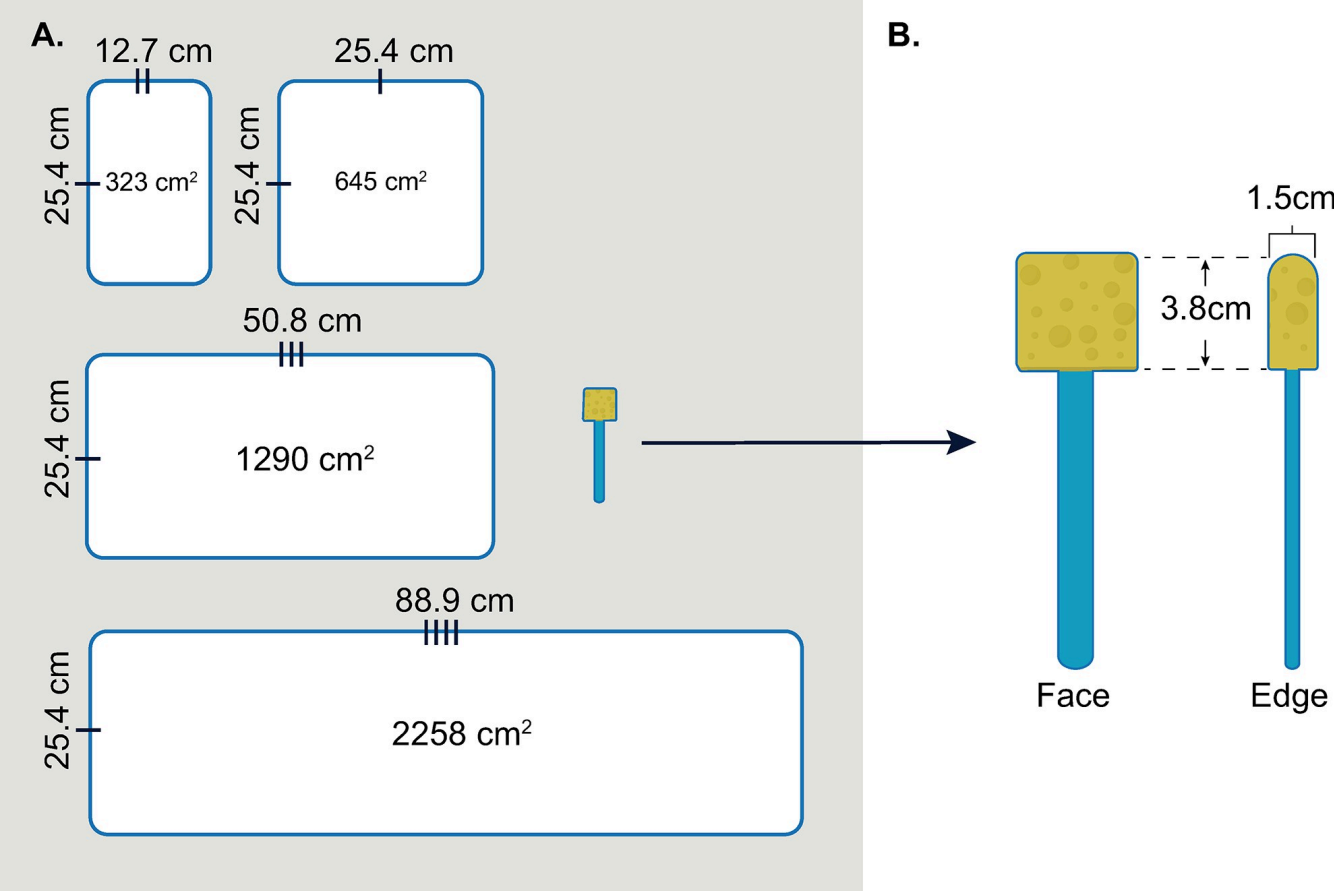
Surface areas and dimensions of coupons and cellulose sponge sampling tool evaluated. (A) Scaled depiction of sampling tool and stainless-steel coupons used for testing. (B) Representative graphic of cellulose sponge tool (sponge) detailing the two different sides of one sponge: a larger “face” (3.8 × 3.8 cm) and a smaller “edge” (3.8 × 1.5 cm).

Our composite sampling approach was similar to the Single Medium, Single Pass Composite sampling method described by Hess et al. [14]; we used a single sampling sponge tool to sample four distinct surface areas (coupons), with a different side of the sponge used for each coupon. Every side of the sponge, including its larger (face) surfaces (2) and smaller (edge) surfaces (2) sampling aspects, underwent evaluation to identify the optimal surface area (OA) for the recovery of each organism (Fig 1). This assessment involved determining the percent recovery (%R), total colony forming units (CFU) and CFU per square centimeter (CFU/cm^2^).

**Fig 2:**
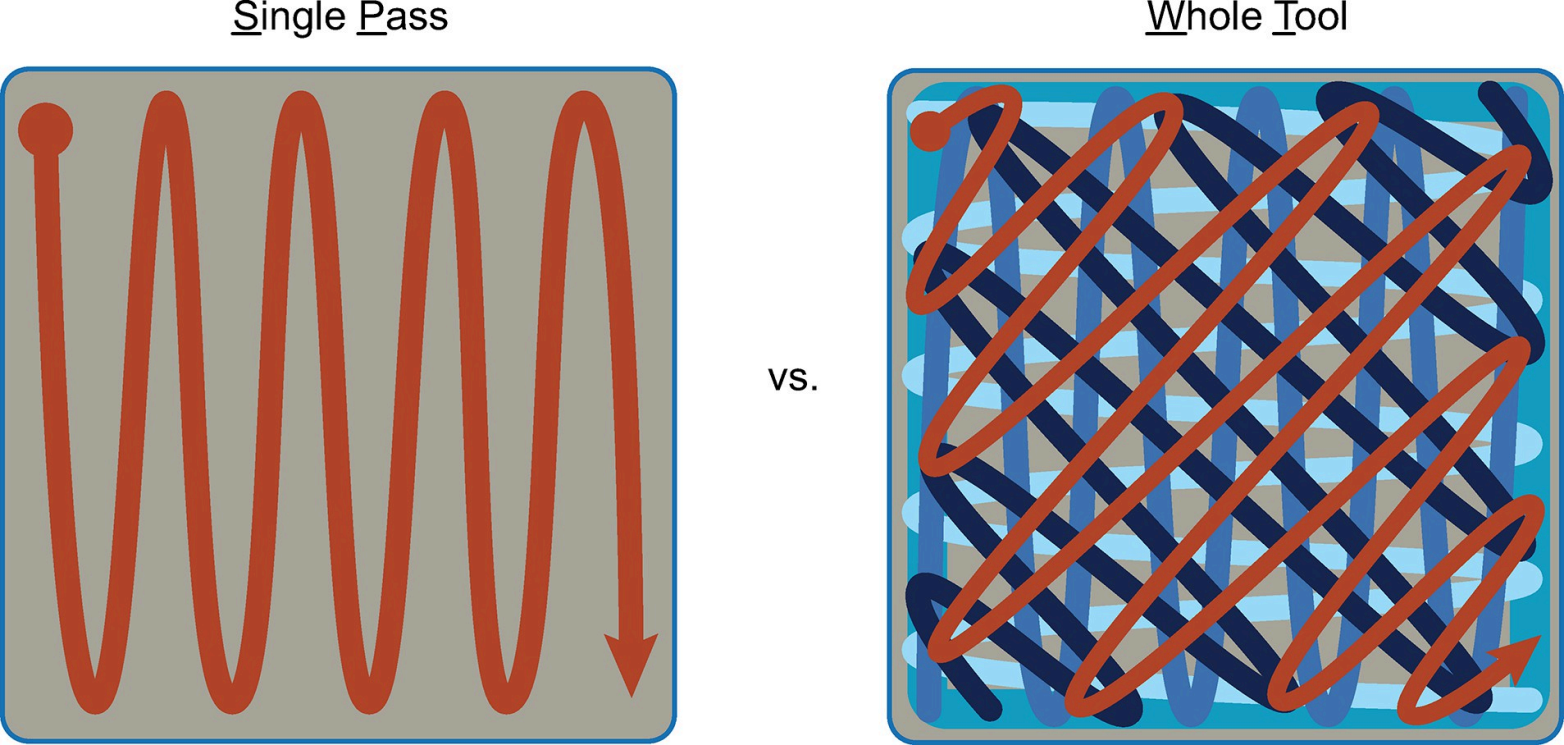
Single pass (SP) and whole tool sampling coverage. (A) Single Pass (SP): using one side of a sponge across independent surface areas with complete coverage. (B) Whole Tool (WT): each side of the sponge is used independently over the same, limited, standard 645 cm^2^ surface area resulting in complete and overlapping coverage.

The National Institute for Occupational Safety and Health (NIOSH) provides guidance for sampling *Bacillus anthracis* spores using a cellulose sponge sampling procedure [15]. For this study we refer to the use of the NIOSH method as the CDC standard whole tool (WT) sampling method. The WT method specifies using all sides of a sponge sequentially on the same 645 cm^2^ surface (Fig 2).

Composite samples were compared with the CDC standard WT sampling method, a method which uses all four sides of the sponge to sample one standard surface area (645 cm^2^) [15]. Additionally, a theoretical composite (TC) was generated based on OA findings and compared with WT sampled results. Experiments were conducted in two phases (Table 1).

**Table 1:** Summary of test parameters.

Phase	Sampling Method	Coupon Sizes (cm^2^)	Analysis	Objectives
**I**	SP^a^	323, 645, 1290 & 2258	1. Greatest %R	Determine OA^d^
	& WT^b^		2. Total CFU	Compare SP of edge or face to CDC method (WT)
**II**	WT & TC^c^	OA for each organism, 645 & 323	1. CFU/cm^2^	1. Construct TC recovery using OA and conservative area (323 cm^2^)
			2. Total CFU	2. Compare TC recovery with WT (645 cm^2^) and multiple WT over same area as TC

^a^ SP: Single pass of the sponge over a surface using edge of sponge or face of sponge.

^b^ WT: Standard CDC method in which all sides of sponge used to sample 645cm^2^.

^c^ TC: "Theoretical Composite" Total CFU determined from recovery obtained from OA for each organism. ^d^ OA: Optimum Area, the area with the greatest amount of %R.

**Phase I: Single pass optimum areas and whole tool comparison.** We investigated the optimum surface area to sample each organism, based on the recovery efficiency measured as %R relative to the seeded inoculum. Four coupons of various area sizes (323, 645, 1290 and 2258 cm^2^) were each inoculated in triplicate with each organism (Fig 1). Both distinct portions of the sponge (face and edge) were used independently to sample coupons with only a single pass (SP) of the sponge (Fig 1B). The %R from each coupon was compared to the %R of the next larger coupon in the series; and if a significant difference was detected between a coupon size, the smaller coupon size was determined to be optimum [14]. The %R and total CFU of each organism and their respective optimum areas were compared to those of a CDC standard method that uses all sides of the sponge to repeatedly sample the same surface 625 cm^2^ area (WT method). Each size of coupon was inoculated and sampled in triplicate for each organism. Recovered organisms were plated in triplicate. This process was repeated on at least three separate days for each organism to obtain a minimum of *n* ≥ 27 data points per organism.

**Phase II: Theoretical composites and comparison to the CDC standard whole tool and multiple whole tool samplings.** Utilizing the OA recovery data from Phase I, a theoretical composite (TC) value was constructed for each organism by calculating their respective colony forming units per square centimeter (CFU/cm^2^).

The median total CFUs from the constructed TCs were compared to the median total CFUs recovered using the WT method from Phase I when sampling 645 cm^2^ only and 2) when sampling the area determined to be the TC for each organism.

### Organisms and inoculum preparation

Five representative AROs were evaluated; *Acinetobacter baumannii* (AB; Multilocus Sequence Type 12), carbapenemase-producing KPC+ *Klebsiella pneumoniae* (KPC; ATCC BAA-1705), vancomycin-resistant *Enterococcus faecalis* (VRE; A 256), methicillin-resistant *Staphylococcus aureus* (MRSA; ATCC 43300) and *Clostridioides difficile* spores (CD; ATCC 43598). Isolates of AB, KPC, VRE and MRSA were cultured on Tryptic Soy Agar with 5% Sheep Blood (BAP; Remel, Lenexa, KS) at 37°C for 18-24h prior to creating the inoculum. The CD spores were prepared as previously described [16]. CD spore titer was confirmed on Brain Heart Infusion Agar with Horse Blood and Taurocholate (BHI-HT; Hardy Diagnostic) and incubated in an anaerobic chamber for 48 h.

Cell or spore suspensions were prepared at a concentration of ~10^8^ CFU/mL in a solution of PBS with 0.02% Tween 80. These suspensions were then diluted stepwise to reach a final concentration of ~10^5^ CFU/mL in Butterfield’s buffer (BB) with 20% Artificial Test Soil (ATS, Healthmark Industries Company, Inc., Frasier MI). Concentration was verified by plate counting. Negative controls consisted of ATS excluding respective test AROs.

### Coupon preparation, inoculation, and sampling

Stainless-steel test coupons (24-gauge, T-304; Stewart Stainless Supply, Suwanee, GA) were cut into four sizes; 323 cm^2^, 645 cm^2^, 1,290 cm^2^ and 2,258 cm^2^ (Fig 1A). Coupons were washed with dilute non-antibacterial soap, triple rinsed with deionized water, rinsed finally with 70% ethanol, air dried, wrapped in kraft paper and autoclave sterilized (gravity cycle 20 min,121.1°C, 15 PSI).

To simulate droplet deposition and enable reproducible inoculation, four droplets of a 10^5^ CFU/mL in 40 μL cell suspensions (total of 160 μL applied per coupon, resulting in ~10^4^ CFU/coupon) were deposited ~3.8 cm from the corner edges of the steel coupons. A 323 cm^2^ coupon inoculated with ATS served as a negative control for each repetition. The droplet of sample inoculum was expanded to ~20 mm using the tip of the depositing pipette. Coupons were left to dry in a closed biological safety cabinet without air flow for two hours, until visibly dry.

Samples were collected using a cellulose sponge tool (sponge) pre-moistened with neutralizing buffer (3M™ Sponge-Stick with neutralizing buffer; 3M, St. Paul, MN). The sponge dimensions are defined in Fig 1B.

### Surface sampling methods and processing

**Fig 3:**
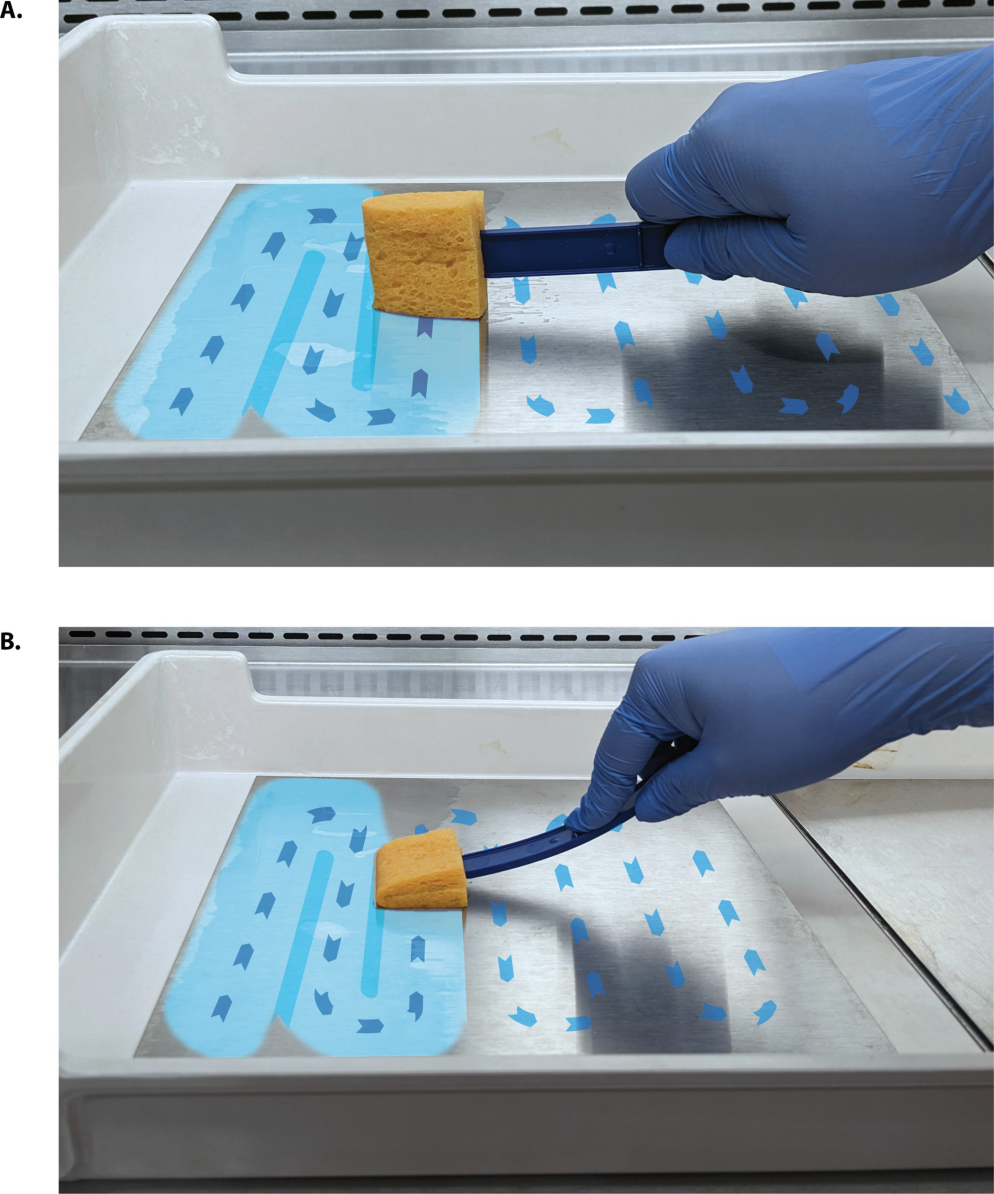
Single pass (SP) sampling method for the sides of a sampling tool. When sampling a surface, the tool’s active side is flush to the coupon and sampling action performed edge-to-edge in a “S” shaped continuous movement over the surface area. (A) Edge utilizes the smaller side of the sponge; (B) Face, utilizes the larger side of the sponge.

Samples were collected using either one of two methods: 1) CDC standard whole tool (WT) method (645cm^2^ only) or 2) a single pass (SP) method using the face or edge of the sponge (Figs 1–3) [15]. For the WT method, the face was selected to wipe a 645 cm^2^ surface area with an edge-to-edge non-overlapping “S” pattern with the sponge face flat onto the surface being sampled (Fig 2) [15]. The sponge was then flipped to the opposite face and the same surface was wiped, perpendicularly, overlapping the previous pattern with the same “S” pattern. The edge of the sponge was then placed in the corner of surface area and wiped diagonally to the opposite corner and again—with the remaining edge perpendicular to the previous “S” pattern in a diagonal direction (Fig 2B) [15]. Finally, the tip of the sponge was used to wipe the perimeter of the sampling area. The sponge was broken from the handle at the score and aseptically placed back into the sealable transport bag.

For the SP method either the face or edge of the sponge was selected based on experimental run and the sampler made one pass over the coupon’s steel surface by wiping in a continuous “S” shaped pattern (once, edge-to-edge pass) over the coupon (Figs 2A and 3). The sponge was broken from the handle at the score and aseptically placed into the sealable transport bag.

### Sample processing and culture methods

Once samples were collected, the sponges were held for one hour at ambient room temperature to simulate adherence during transport. The sponges were aseptically removed from the plastic handle remnants and placed into sterile sealable bags (Stomacher® 400 Circulator Bags; Seward, Bohemia, NY) filled with 45 mL of phosphate-buffered saline containing 0.02% polysorbate 80 (PBST). Processing followed the American Society for Testing and Materials (ASTM) E3226-19 method, which was also used in other studies [2, 17, 18]. Samples were homogenized using a paddle blender (Stomacher® 400 Circulator; Seward, Bohemia, NY) for one minute at speeds optimum for each organism based on internal evaluations (AB, 200; CD, 260; KPC, 200; MRSA, 260; and VRE, 200 RPM). Excess liquid was expressed from the sponge and the eluate concentrated by centrifugation at 2,700 x g for 20 minutes. All but ~ 6mL of supernatant was discarded, the pellet was resuspended, and the eluate volume was measured and recorded.

Samples were 10-fold diluted in series and plated onto appropriate agar plates; BAP or BHI-HT plates in triplicate. Based on preliminary recovery testing, some of the organisms tested resulted in low recovery CFU counts, typically fewer than 25 CFUs per mL of eluate. In these cases, 1mL aliquots were vacuum filtered through 0.45um mixed cellulose ester filters (Microfunnel, Pall Corp., Ann Arbor, MI) at ~17–20 PSI to capture cells on the filters. The filters were placed on appropriate culture media plates (BAP or BHI-HT). The plates with vegetative cells were incubated at 36°C, aerobically for 18–24 hours, plates with CD spores were incubated in an anaerobic chamber at 36°C for 48 hours. Colonies on plates and filters were counted and recorded using the Flash and Grow™ Automatic Colony Counter (Neu-tec Group Inc., Farmingdale, NY).

### Calculations, statistical analysis, and data visualization

The total CFU was recorded for each variable (organism, sponge side [face or edge] and surface area), compared to the inoculum deposited to calculate %R and used to measure median %R and CFU/cm^2^. Each variable was inoculated and sampled in triplicate sets with triplicate plates. As seen in Tables 2 and 3 —n for each variable was ≥27. The OA for each organism were designated if a significant lower median %R was seen between adjacent coupon sizes, the smaller size was chosen as the optimum. TC for the optimum areas were created by doubling the median total CFU of each organism recovered using a SP of both the face and edge of the sponge and summed together, as seen in Eq 1.


Recovery=2×((Face)+(Edge)) (1)

**Table 2:** Percent recovery of healthcare-associated pathogens from stainless steel surfaces using a single pass of a cellulose sponge.

	Surface Area (cm^2^)	Single Pass Face			Single Pass Edge		
		n	Median	IQR^a^	n	Median	IQR^a^
Gram-negative			(%)	(±%)		(%)	(±%)
*Acinetobacter baumannii*	**323**	30	6.68	2.75	30	4.34	2.75
(AB)	**645**	27	9.50	4.00	27	3.05	4.00
	1290	34	4.64	3.00	33	1.45	1.00
	2258	30	4.23	2.00	35	0.51	1.00
*Klebsiella pneumoniae*	323	30	2.4	1.00	30	3.21	4.75
(KP)	**645**	27	3.48	2.00	30	1.09	3.75
	**1290**	27	2.87	3.00	29	0.69	4.00
	2258	30	0.52	1.00	29	0.1	0.00
**Gram-positive**	** **	** **	** **	** **	** **	** **	** **
methicillin-resistant *Staphylococcus aureus*	**323**	36	8.14	7.25	27	9.78	6.50
(MRSA)	645	36	5.23	4.00	27	5.01	8.00
	1290	36	4.36	3.00	27	4.98	2.00
	2258	36	4.26	4.25	27	4.67	2.50
*Enterococcus faecalis*	**323**	27	14.95	14.00	30	12.09	5.75
(VRE)	645	30	10.69	10.50	30	6.4	5.00
	1290	27	8.01	4.00	27	3.94	2.00
	2258	27	8.55	6.00	27	4.9	2.00
**Spore former, Gram-positive**	** **	** **	** **	** **	** **	** **	** **
*Clostridioides difficile*	323	27	41.51	21.50	27	21.02	5.50
(CD)	**645**	27	37.31	11.50	27	14.64	6.50
	1290	27	31.64	10.50	27	15	6.00
	2258	27	24.75	4.00	27	12.7	7.50

*n* represents the number of recovery culture plates.

^a^ IQR = Interquartile Range

Highlighted rows represent selected Optimized Area (OA).

**Table 3:** Median percent recovery, total CFU, and CFU/cm^2^ of healthcare-associated pathogens from stainless steel surfaces using single pass or WT of a cellulose sponge.

	Method	n	OA (cm^2^)	Median %R ± IQR^b^ (%)		P-value^a^ Face vs WT (%R)	Median Total CFU ± IQR		P-value^a^ Face vs WT (Total CFU)	Median CFU/cm^2^ ± IQR		P-value^a^ Face vs WT (CFU/cm^2^)
Gram-negative												
*Acinetobacter baumannii* (AB)	Edge	27	323	2.22	2.00		2.46E+03	1.85E+03		7.64	5.71	
	Face	27	645	3.69	3.00	0.15	4.32E+03	3.05E+03	0.20	6.70	4.71	0.20
	WT	27	645	5.76	4.50		4.76E+03	1.39E+03		7.37	2.15	
*Klebsiella pneumoniae* (KP)	Edge	27	645	0.40	1.00		5.38E+02	4.78E+02		1.67	1.49	
	Face	27	1290	0.56	1.00	0.55	6.19E+02	4.98E+02	0.64	0.48	0.39	p < 0.05
	WT	35	645	0.50	1.00		8.13E+02	6.12E+02		1.26	0.09	
**Gram-positive**												** **
Methicillin-resistant *Staphylococcus aureus* (MRSA)	Edge	27	323	18.91	7.00		7.31E+03	7.97E+03		22.70	24.70	
	Face	27	323	40.81	10.00	0.10	1.66E+04	1.64E+04	0.43	51.60	50.75	p < 0.05
	WT	27	645	44.85	7.00		1.83E+04	1.66E+04		28.40	25.76	
*Enterococcus faecalis* (VRE)	Edge	33	323	19.11	9.00		1.35E+04	1.08E+04		41.70	33.30	
	Face	33	323	37.37	15.00	0.08	2.64E+04	1.66E+04	0.20	81.80	46.90	p < 0.05
	WT	33	645	39.25	18.00		2.76E+04	2.05E+04		42.80	31.80	
**Spore former, Gram-positive**												** **
*Clostridioides difficile* (CD)	Edge	27	645	32.76	12.50		4.80E+03	3.62E+04		7.44	56.05	
	Face	27	645	53.60	19.00	p < 0.05	9.69E+03	6.58E+04	0.26	15.00	102.15	0.25
	WT	27	645	63.63	16.50		1.09E+04	7.05E+04		17.00	109.35	

*n* represents the number of recovery culture plates. Data compared using the Wilcoxon Rank Sum Test.

^a^ Significance threshold: P-value represented in this figure (*p* = 0.05) was calculated using a non-parametric one-sided approach, where a higher observed value is considered more significant (Pr > Z).

^b^ IQR = Interquartile Range

For optimum surface area for an unknown target organism, we investigated a conservative approach to composite sampling by sampling the smallest surface area (323 cm^2^) with both the face and edge of the sponge for all organisms. The conservative TC was calculated for each organism using Eq 1 whereby the values used in the calculation were the median total CFU recovered from the 323 cm^2^ coupons, resulting in a total area of 1,290 cm^2^ [1]. Multiple WT and conservative multiple WT recovery was determined by obtaining the CFU/cm^2^ for the standard WT sampling area and multiplying by either the optimum TC area for each organism or the conservative TC area. For the conservative multiple WT recovery, the recovery from a single WT was doubled (2 × 645 cm^2^) to match the total area for conservative TC (1,290 cm^2^). These data were used to compare the WT, multiple WT, TC, conservative TC, and conservative multiple WT methods for each organism.

Statistical analyses were using SAS software, Version 9.4 of the SAS System for PC. Copyright © 2019 SAS Institute Inc. SAS Institute Inc., Cary, NC, USA.). Figures and additional data output were generated using R Statistical Computing Software version 3.53 using the dplyr 1.0.5 and ggplot2 3.3.3 packages [19]. Due to the asymmetric CFU distribution we opted to use non-parametric tests as these are distribution-free and less subject to extreme outliers. Non-parametric comparisons for OA were evaluated using Mood’s median test to assess significant differences between median recoveries. Non-parametric comparisons comparing sampling approach methods (face, edge, and WT) were evaluated using the Wilcoxon signed rank to assess significant differences between median recoveries. Statistical significance was set at p ≤ 0.05.

## Results

### Phase I: Single pass optimum areas and whole tool comparison

**Fig 4:**
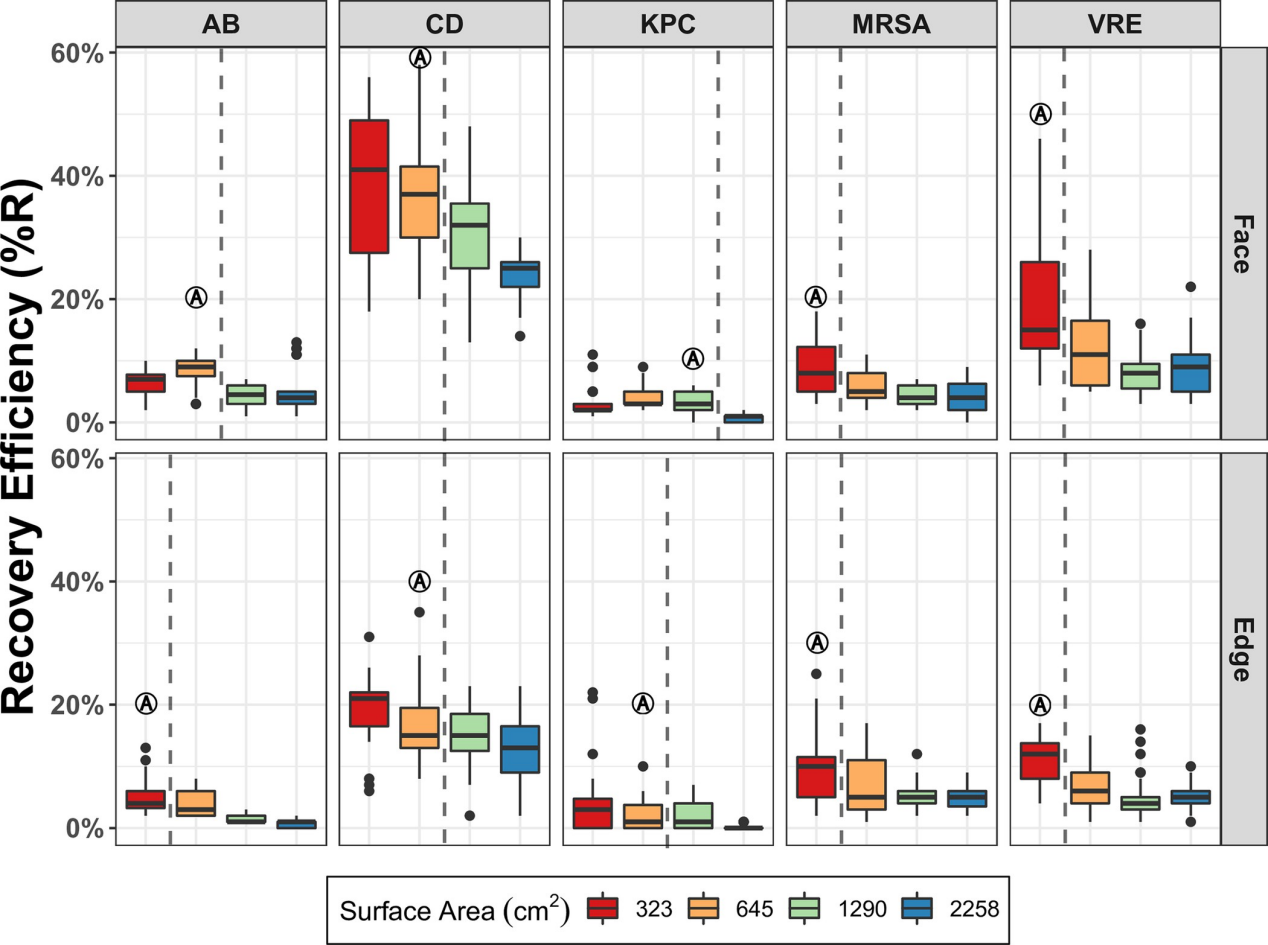
Median recovery efficiency (%R) of five healthcare-associated pathogens using a single pass sampling with either face or edge of a sponge over four surface areas (cm^2^). Five AROs represented: carbapenemase-producing KPC+ *Klebsiella pneumoniae* (KPC), *Acinetobacter baumannii* (AB), methicillin-resistant *Staphylococcus aureus* (MRSA), vancomycin-resistant *Enterococcus faecalis* (VRE) and *Clostridioides difficile* (CD) spores. The dotted line indicates a significant difference in pathogen recovery between the different coupon sizes. “A” noted boxes designate the selected optimum area (OA) for each organism. Minimum n = 27, maximum n = 36.

**Fig 5:**
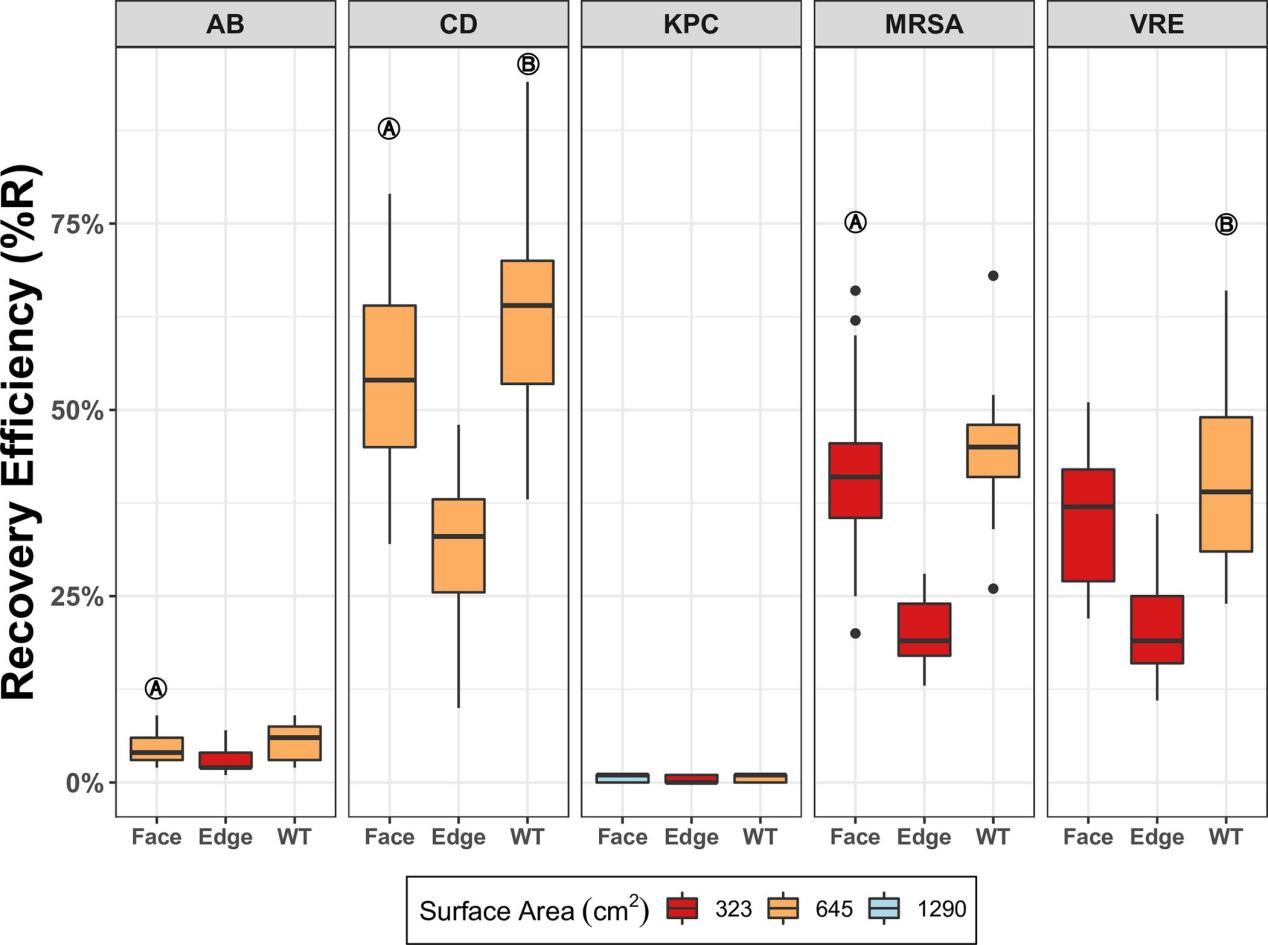
Median recovery efficiency (%R) using either the face or edge on each organism’s SP optimum area or the WT method. Five AROs represented: carbapenemase-producing KPC+ *Klebsiella pneumoniae* (KPC), *Acinetobacter baumannii* (AB), methicillin-resistant *Staphylococcus aureus* (MRSA), vancomycin-resistant *Enterococcus faecalis* (VRE) and *Clostridioides difficile* (CD) spores. Surface areas for face and edge depicted here are the optimum areas (OA), determined for each organism in the first part of this study. SP single pass of one sponge’s side across a surface; OA, optimum surface area determined for each organism; WT, standard CDC method in which all sides of the sponge are sequentially used to sample one 645 cm2 surface. “A” noted boxes represent significant difference (p<0.05) detected between face and edge %R. “B” noted boxes represent significant difference detected between face and WT %R. Sample sizes ranged from a minimum n = 27 to a maximum n = 35.

The median %R varied between organisms, although for all organisms the %R generally decreased as the surface area sampled increased. For all organisms the median %R from the largest coupons size (2,258 cm^2^) was significantly lower than other coupon sizes for both the face and edge evaluations (Table 2 and Fig 4). Therefore, 2,258 cm^2^ was not chosen as an optimum surface area (OA) for any organism (Table 2 and Fig 4). For most organisms, the %R when using the face was greater than the %R obtained using the edge at equivalent sampling areas (Table 2). The exception to this was KPC, for which no significant differences were seen between face or edge (*p* = 0.75) (Table 2 and Fig 5).

The OA selected varied between organisms. For most organisms tested, the OA when using the edge of the sponge was the smallest sized coupon (323 cm^2^). However, for the test organisms KP and CD, the edge OA selected was larger (645cm^2^). In contrast, the OA for the face of the sponge ranged from 323 cm^2^ to 1,290 cm^2^ (Table 2 and Fig 4).

When we compared the recovery using only the face of the sponge to recover organisms from their respective OA to the use of the WT on the standard CDC WT area (645 cm^2^) we found no significant differences in %R for any organism, except for CD (Fig 5 and Table 3). Notably, the OA for CD was found to be the same size as the WT sampling area (Fig 5 and Table 3). When analyzed as median total CFU recovered, there was no significant difference between the OA and the WT method for any organism (Table 3). When analyzed as CFU/cm^2^ three organisms showed significant differences between OA and WT method; KPC, MRSA and VRE (Table 3), while AB and CD CFU/cm^2^ recovery showed no differences (Table 3). Notably, the OA for KPC was larger than the WT sampling area and both MRSA and VRE OAs were smaller than the WT sampling area (Table 3).

### Phase II: Theoretical composites and comparison to the CDC standard whole tool and multiple whole tool samplings

Theoretical composites (TC) for each organism were calculated using the total CFU recovered from their respective OA. Because the OAs were different, the TC areas differed and were determined to be (based on Eq 1) 1,290 cm^2^ for *S*. *aureus* and *E*. *faecalis*, 1,936 cm^2^ for *A*. *baumannii*, 2,580 cm^2^ for *C*. *difficile* spores and 3,870 cm^2^ for *K*. *pneumoniae* (S1 Table). Note that the smallest area covered by a TC sampling (1,292 cm^2^) is still double the area sampled using the standard CDC WT method, 645 cm^2^ (S1A Table).

**Fig 6:**
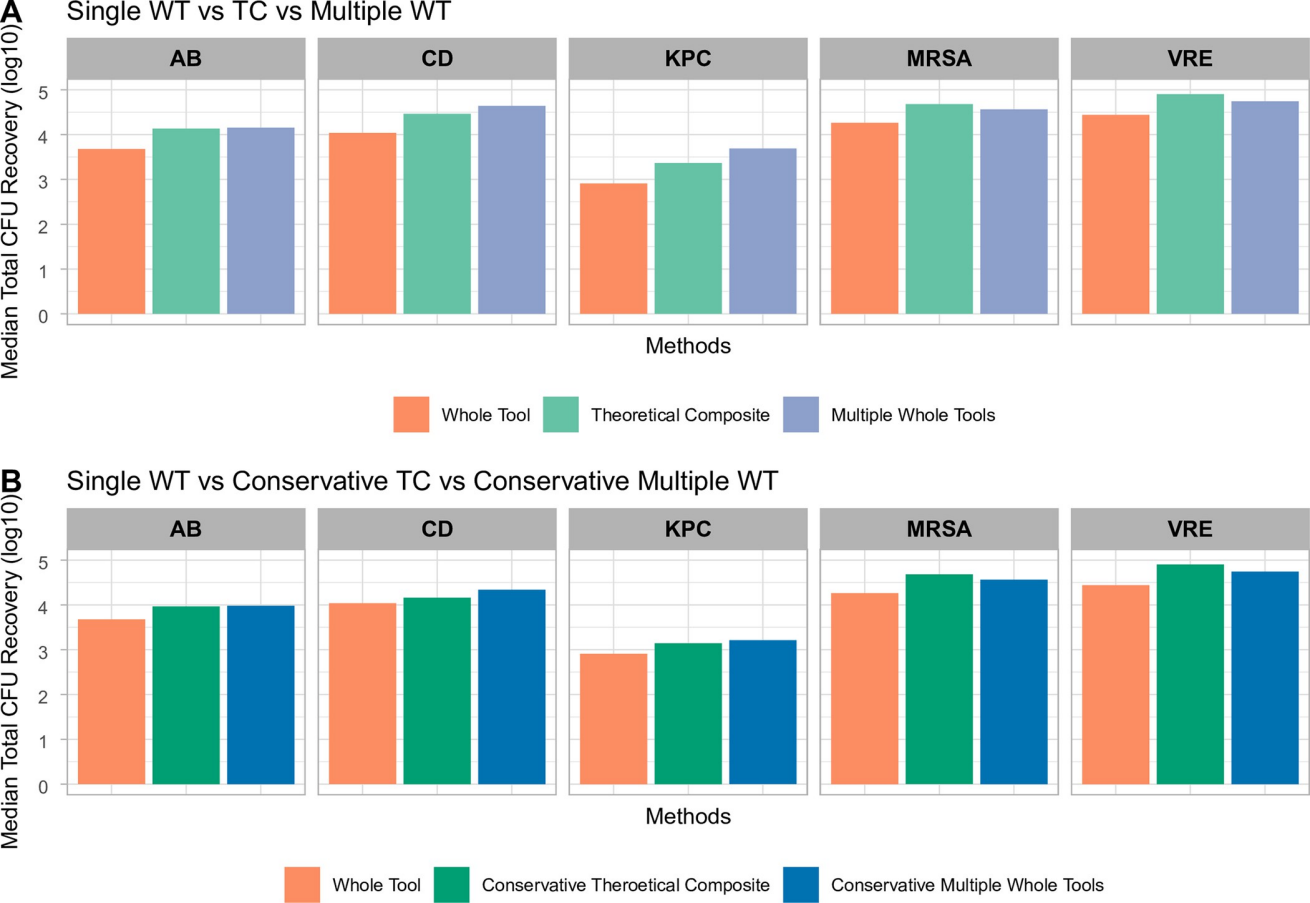
Median total CFU recovered: Theoretical composites (TC) vs. whole tool (WT) method. Five AROs represented: carbapenemase-producing KPC+ *Klebsiella pneumoniae* (KPC), *Acinetobacter baumannii* (AB), methicillin-resistant *Staphylococcus aureus* (MRSA), vancomycin-resistant *Enterococcus faecalis* (VRE) and *Clostridioides difficile* (CD) spores. Optimum areas (OA) are different for each organism, determined in Phase I of this study. Single Whole Tool (WT) is the recovery from the use of all sides of a single sponge over a 645 cm^2^ surface area. Theoretical composite (TC) was calculated by (2 × OA recovery with face) + (2 × OA recovery with edge). Multiple WT is comprised of the median total CFU for a 645 cm^2^ adjusted to the same area as a given organism’s TC area. Conservative TC was calculated by (2 × 323 cm^2^ recovery with face) + (2 × 323 cm^2^ recovery with edge). Conservative Multiple WT is the median total CFU for 645 cm^2^ adjusted to 1290 cm^2^. (A) Single WT vs. TC vs. Multiple WT. (B) Single WT vs. Conservative TC vs. Conservative Multiple WT. Minimum n = 27; maximum n = 36.

For all organisms, the total CFU recovered by the TC sampling (all ≥ 1290 cm^2^) was found to be greater than the median total CFU recovered when using the CDC standard WT (645 cm^2^ only) method (Fig 6A and S1 Table). The TC total CFU recovery for organisms MRSA and VRE was shown to be greater than if using multiple WT samplings over their OA of 1290 cfu/cm^2^ (Fig 6A and S1 Table). While the total CFU recovered for AB, KPC and CD was greater with multiple WT samplings, the total CFU for each method were within the same order of magnitude (same log_10_ CFU) as the TC recovery (Fig 6A and S1 Table).

The comparisons of total CFU recovered using our conservative TC approach (over the conservative [1,290 cm^2^] area) and those using the standard WT (645 cm^2^) or conservative multiple WT samplings can be seen in Fig 6B and S2 Table. Both conservative TC and multiple WT sampling approaches provide a greater total CFU than the standard WT (Fig 6B and S2B Table). The conservative TC total CFU was greater for organisms MRSA and VRE than conservative multiple WT samplings over the same surface area (Fig 6B and S2 Table). While the total CFU recovered for AB, KPC, and CD was greater with conservative multiple WT samplings over the same conservative area, the total CFU for each method were within the same order of magnitude (same log_10_ CFU) as the conservative TC recovery (Fig 6B and S2 Table).

## Discussion

We demonstrate in this work that, with few exceptions, using only the face of the sponge on the designated optimum surface areas yields a lower, but not a significant difference in %R (exception CD), total CFU recovery and CFU/cm^2^ (exception MRSA and VRE) than using the WT on the standard 645 cm^2^ area (Fig 5 and Table 3). The remaining three sides of the sponge are additional opportunities to collect more pathogens from additional sampling areas using our composite sampling approach, thus expanding sampling surface area limits and conserving additional sponge use.

No sampling method is capable of recovering 100% of the organisms present on a surface [20]. The outcome of environmental sampling is influenced by many factors including: the surface area and material sampled, sampling tool selection, premoistening agent, target organism characteristics, organic substances present, sampling method consistency (pressure during sampling and number of passes over a sample area) and laboratory processing and detection methods [2, 10, 15, 18, 20–22].

Composite sampling adds additional complexity to the variability inherent in environmental surface sampling by combining multiple surfaces or samples [2, 8, 9, 23]. Tufts et al. [24] and Hess et al. [14] have explored composite approaches for surface sampling, specifically for spores of the *Bacillus anthracis* surrogate *Bacillus atrophaeus*.

To better understand how composite sampling would compare to a standard (non-composite) method, we controlled for individual variables (sampling tool, method of sampling, sample deposition and organism) and the limitations of surface area were examined. We focused exclusively on stainless-steel surfaces due to common use in healthcare settings and prior use of the material in other sampling efficiency studies [24–27].

We chose the premoistened cellulose sponge stick for this evaluation, as it is commonly used for healthcare environmental studies and public health investigations, is well characterized in a validation study for use for *Bacillus anthracis* response and cited in a standard test method for processing available from the ASTM [17, 23, 24, 27–30]. The design of the sponge includes significantly larger functional surfaces for sampling than a swab, enabling recovery of organisms from larger surface areas. Unlike a swab, a sponge has four potential sampling surfaces consisting of two faces and two edges (Fig 1).

A trend of descending %R emerged as sampling area size increased. The sponges may be collecting but not retaining the organisms when sampling, as demonstrated by Tufts et al. with *Bacillus atrophaeus* spores [24]. Though the Tufts et al. experimental design differed from ours, they did note 28% loss of spores when using one sponge across four 929cm^2^ surfaces [24]. The impact of sampling area size on %R was also seen in a study evaluating swabs when sampling healthcare pathogens [13].

Our findings showed that for most organisms, the %R was greater when sampling with the face than when sampling with the edge of the sponge, which suggests that a larger sampling tool enables more organisms to be recovered. This does not eliminate the need for swabs or other smaller sampling tools for situational uses, such as small or hard to reach surfaces, or unique reservoirs.

Variation in the %R for each organism may result from differences in bacterial persistence when dried on fomites, as well as the unique adherence properties of each organism to steel or to the sampling sponge, as discussed in Rose et al. [18]. Lemmen et al. also found that MRSA and VRE were recovered at higher %R than KPC and AB and surmised that Gram positive bacteria persisted longer than Gram negative bacteria [31]. The strain of VRE used in this study was previously found to persist beyond 28 days when held at 26°C and 57% relative humidity [32]. Other strains of KPC have been cultured from the environment for extended time periods, with one study reporting persistence up to 20 months [25, 26, 31, 33]. Under the same conditions of this study, this same strain of KPC was reported to decline rapidly in cultivability after deposition (5 log_10_ reduction within 5 days) possibly contributing to difficulty in detection by culture [25, 26, 34]. KPC recovery was lower than all other organisms evaluated, regardless of analysis, as %R or total CFU. This may be due to reduced persistence or increased adherence to the sampling surface or sampling tool as compared to other organisms [32, 34–36].

Factors that influence an organism’s adherence to a surface can include the material’s hydrophobicity, physicochemical properties, and presence of organic matter [18, 37–41]. In addition, the cell characteristics, such as cell or spore hydrophobicity and charge, the presence of flagella or fimbriae and the production of extracellular polysaccharides can also influence adherence [36, 42–45]. *Bacillus* and *Clostridioides* spores are more hydrophobic than their respective vegetative cells, a trait correlated with less adherence to a surface, this may explain the greater %R over surface areas compared to the pathogens observed in this study [46].

The Tufts et al. work compared cellulose sponge sampling with a single swipe to the WT method from a 929 cm^2^ steel surface and found no significant difference in total CFU recovered [24]. In contrast, Hess et al. compared a SP method over four inoculated steel coupons (645 cm^2^ each) to using one WT multiple times across all four inoculated coupons and found that multiple WT samplings of the four surfaces recovered more spores than four individual passes with each side of one sponge tool [14]. Differences in study design between these two investigations and the current work are many, including inoculum size (Tufts: 10^7^ spores per coupon, Hess: 10^2^ spores per coupon) and inoculation method (Tufts: aerosol deposition; Hess: liquid deposition) [14, 24]. We should note that the Hess composite investigation included an approach not investigated here; the post-sampling composite approach [14]. Post-sampling compositing included using multiple sponges and the standard CDC 645cm^2^ coverage area for up to eight surfaces, then processing each sequentially in the laboratory, in the same elution buffer/stomacher bag [14]. This was found to enable the best recovery within this evaluation [14]. Yet, this approach does not reduce time in the field and minimally reduces time in the laboratory; in fact, this approach may increase the chance of contaminating the sample by requiring more manipulation of the sponges and their containment bags during processing. Due to these aforementioned factors, we did not include this method in our study.

Our composite sampling approach considers the optimum sampling areas determined statistically at the beginning of this study. Depending on the organism, the composite approach can increase the total sampling surface area by 2, 3 or 4 times that of the standard CDC WT method, which will also save the number of sponges used for a given sampling event (S1 Table) and therefore save lab time and resources processing and culturing organisms from the sponges.

If the target organism or optimum area for an organism is not known and time and resources are limited, we suggest a conservative composite sampling approach—with the understanding that for some organisms it may not be as sensitive as the standard CDC WT method. This conservative approach limits the sampling area for each of the sponge’s four sides (2 x face and 2 x edge) to 323 cm^2^ (S2 Table). This approach results in a total sampling area of 1290 cm^2^ which is still twice the recommended surface area for the standard CDC WT method (S2 Table).

This conservative approach considers the concept of diminishing recovery with increased surface area. While smaller surfaces may not provide a statistically better recovery for an untested organism, the smaller sampling areas provide equivalent or greater percent recovery for most of the tested organisms (S2B Table). This can be seen in the results from the percent recovery comparisons between the optimized CFU recovery to the conservative CFU recovery in S1B and S2B Tables.

Percent recovery increased for organisms AB, KP and SA when using the conservative approach, likely due to the larger optimized surface sampling area (S1B and S2B Tables). Percent recovery for VRE, SA and CD remaining virtually unchanged or very close (1% increase for SA), expected for VRE and SA since the optimized and conservative areas are the same. It is interesting that CD maintains its percent recovery comparison even as the conservative surface area is smaller than the optimized area (S1B and S2B Tables). The data trends demonstrate a decline in recovery as the sample area increases, reenforcing the impact that sampling area affects recovery.

The recovery of organisms in our Phase II study assumes equal numbers of organisms on each surface sampled, which may not be the case in a healthcare setting, but our investigation illustrates the potential for recovery of bacteria given various sampling areas. We also recognize that healthcare surfaces may contain multiple organisms, and the presence of non-target organisms may influence the recovery of target organisms, a concept not included in this investigation. This study did not investigate the detection of organisms by molecular means and it is possible that the conclusions of optimum areas, %R or total recovery may be different if detection methods other than culture are employed.

## Conclusions

To date, few studies have investigated composite surface sampling of healthcare pathogens. We observed that as the sampling increased, most organisms experienced a decline in recovery, regardless of the sponge side used. The loss varied among each organism, leading us to identify the optimum surface area for each.

The findings indicate that using a single side (face) of a sponge yields comparable recovery to using all sides over the same area, suggesting the initial pass recovers most organisms. Thus, we propose a composite method using the different sponge sides, independently, for four separate surface areas.

This composite approach is practical and cost-effective while potentially recovering more pathogens than the CDC method by increasing allowed sampling surfaces. For organisms like KPC, there is a risk of lower recovery, but this may be an acceptable loss if contamination is high enough for detection and the target organism is known. The decision to use this method should align with the sampling event’s goal.

For organisms tested, optimized areas for best recovery are 1290 cm^2^ for *S*. *aureus* and *E*. *faecalis*, 1936 cm^2^ for *A*. *baumannii*, 2580 cm^2^ for *C*. *difficile* spores and 3,870 cm^2^ for *K*. *pneumoniae*. When the optimum area is unknown, a conservative using 323cm^2^ per sponge side (1290 cm^2^ total) is recommended. This conservative area, double the standard CDC standard WT method (645 cm^2^), reduces the number of tools and supplies needed, saving time in sampling and processing.

These composite approaches will enable reduced time to reporting results, enhanced rapid intervention, reduced transmission, prevention of illness and lives saved.

## Supporting information

S1 Table Summary of TC and WT. A. Optimum areas determined for each pathogen in Phase I, standard WT method areas, theoretical composite (TC) areas, and ratio of tools required. ^a^ Standardized Whole Tool Area of 645 cm2. ^b^ Calculated with determined OAs using Eq 1: (2 × (face + edge)). ^c^ Tools Required (WT) represents the ratio of the number of individual sampling tools required for sampling the optimum area if the WT technique (645 cm^2^) has been applied compared to the number of tools needed when optimized for a particular organism using the TC method (optimized TC area unique to the organism). For example, a ratio of 2:1 means that two tools are needed for the WT method compared to one tool for the optimized TC method. NOTE: This demonstrates the number of tools required to cover the optimized area. B. Median CFU recovery of each pathogen: standard WT method, theoretical composite, and multiple WT samplings using the optimum areas determined in Phase I. ^a^ Median CFU from standardized whole tool area of 645 cm2 as determined in Phase I. ^b^ Calculated with median CFU recovered from OAs using Eq 1: (2 × (face + edge)). ^c^ Calculated CFU (standard WT × tools needed for same area as TC method in S1A Table). ^d^ Recovery comparison represents the percentage of recovery of the TC from the recovery of multiple WT calculated by: ((TC / multiple WT) ×100). NOTE: Both multiple WT samplings and TC are comparing recovery over the same OA surface areas for respective organisms.
(DOCX)

S2 Table Summary of conservative TC and WT. A. Conservative sampling areas for each pathogen, standard WT method area, conservative theoretical composite (TC) areas, and ratio of tools required. ^a^ Conservative Area of 323 cm^2^ is the smallest area coupon tested in this study. ^b^ Standardized Whole Tool Area of 645 cm^2^. ^c^ Conservative TC area is 1,290 cm^2^; calculated using the conservative area value of 323 cm^2^ in Eq 1: (2 × (face + edge)). ^d^ Tools Required (WT) represents the ratio of the number of sampling tools required for sampling the optimum area using the WT technique (645 cm^2^) compared to the number of tools needed using the conservative TC method (1,290 cm^2^). For example, a ratio of 2:1 means that two tools are needed for the WT method compared to one tool for the conservative TC method. NOTE: This demonstrates the number of tools required to cover the conservative TC area. B. Median CFU recovery of each pathogen: standard WT method as compared to conservative theoretical composite and conservative multiple WT samplings using a conservative area of 1,290 cm2. ^a^ Median CFU from standardized whole tool area of 645 cm^2^ determined in Phase I. ^b^ Calculated using Eq 1, (2 × (face + edge)) using median CFU recovered from conservative area. ^c^ Calculated CFU (Standard WT x tools needed for same area as conservative TC method in S2A Table). ^d^ Recovery comparison represents the percentage of recovery of the conservative TC from the recovery of conservative multiple WT samplings calculated by: ((conservative TC / conservative multiple WT) × 100). * Denotes organisms whereby the TC area is equivalent to the conservative TC area. NOTE: Both conservative multiple WT samplings and conservative TC are comparing recovery over the same surface areas (1290cm^2^).
(DOCX)

S1 Data Supplementary raw data file: Composite recovery. This file provides raw data on inoculum concentrations and recovery CFUs, detailing surface areas and sampling methods for organisms investigated in the composite study.
(XLSX)
