# Supplementary material for: Investigating surface area and recovery efficiency of healthcare-associated pathogens to optimize composite environmental sampling
Source: PLoS One. 2024 Nov 8;19(11):e0310283. doi: 10.1371/journal.pone.0310283 (PMC11548722; doi:10.1371/journal.pone.0310283)
Supplement: S1 Table — A. Optimum areas determined for each pathogen in Phase I, standard WT method areas, theoretical composite (TC) areas, and ratio of tools required. a Standardized Whole Tool Area of 645 cm2. b Calculated with determined OAs using Eq 1: (2 × (face + edge)). c Tools Required (WT) represents the ratio of the number of individual sampling tools required for sampling the optimum area if the WT technique (645 cm2) has been applied compared to the number of tools needed when optimized for a particular organism using the TC method (optimized TC area unique to the organism). For example, a ratio of 2:1 means that two tools are needed for the WT method compared to one tool for the optimized TC method. NOTE: This demonstrates the number of tools required to cover the optimized area. B. Median CFU recovery of each pathogen: standard WT method, theoretical composite, and multiple WT samplings using the optimum areas determined in Phase I. a Median CFU from standardized whole tool area of 645 cm2 as determined in Phase I. b Calculated with median CFU recovered from OAs using Eq 1: (2 × (face + edge)). c Calculated CFU (standard WT × tools needed for same area as TC method in S1A Table). d Recovery comparison represents the percentage of recovery of the TC from the recovery of multiple WT calculated by: ((TC / multiple WT) ×100). NOTE: Both multiple WT samplings and TC are comparing recovery over the same OA surface areas for respective organisms. (DOCX) [file pone.0310283.s001.docx]

**S1 Table. Summary of TC and WT.**

**A. Optimum areas determined for each pathogen in Phase I, standard WT method areas, theoretical composite (TC) areas, and ratio of tools required.**

|  | **OA (cm^2^)** | | **WT Area**^a^ | **TC Area**^b^ | **Tools Required (WT)**^c^ |
| --- | --- | --- | --- | --- | --- |
|  | **Face** | **Edge** |  |  |  |
| **Gram-negative** |  |  |  |  |  |
| *Acinetobacter baumannii* | 645 | 323 | 645 | 1,936 | **3:1** |
| *Klebsiella pneumoniae* | 1,290 | 645 | 645 | 3,870 | **6:1** |
| **Gram-positive** |  |  |  |  |  |
| methicillin-resistant *Staphylococcus aureus* | 323 | 323 | 645 | 1,290 | **2:1** |
| *Enterococcus faecalis* | 323 | 323 | 645 | 1,290 | **2:1** |
| **Spore former, Gram-positive** |  |  |  |  |  |
| *Clostridioides difficile* | 645 | 645 | 645 | 2,580 | **4:1** |

^a^ Standardized Whole Tool Area of 645 cm^2^.

^b^ Calculated with determined OAs using Equation 1: (2 × (face + edge)).

^c^ Tools Required (WT) represents the ratio of the number of individual sampling tools required for sampling the optimum area if the WT technique (645 cm²) has been applied compared to the number of tools needed when optimized for a particular organism using the TC method (optimized TC area unique to the organism). For example, a ratio of 2:1 means that two tools are needed for the WT method compared to one tool for the optimized TC method.

NOTE: This demonstrates the number of tools required to cover the optimized area.

**B. Median CFU recovery of each pathogen: standard WT method, theoretical composite, and multiple WT samplings using the optimum areas determined in Phase I.**

|  | **Standard WT**^a^ | **TC**^b^ | **Multiple WT Samplings**^c^ | **Recovery Comparison**^d^ (%) |
| --- | --- | --- | --- | --- |
| **Gram-negative** |  |  |  |  |
| *Acinetobacter baumannii* | 4,760 | 13,560 | 14,287 | 95 |
| *Klebsiella pneumoniae* | 813 | 2,314 | 4,878 | 47 |
| **Gram-positive** |  |  |  |  |
| methicillin-resistant *Staphylococcus aureus* | 18,318 | 47,820 | 36,657 | 130 |
| *Enterococcus faecalis* | 27,600 | 79,800 | 55,286 | 144 |
| **Spore former, Gram-positive** |  |  |  |  |
| *Clostridioides difficile* | 10,900 | 28,980 | 43,600 | 66 |

^a^ Median CFU from standardized whole tool area of 645 cm^2^ as determined in Phase I.

^b^ Calculated with median CFU recovered from OAs using Equation 1: (2 × (face + edge)).

^c^ Calculated CFU (standard WT × tools needed for same area as TC method in table S1A).

^d^ Recovery comparison represents the percentage of recovery of the TC from the recovery of multiple WT calculated by: ((TC / multiple WT) ×100).

NOTE: Both multiple WT samplings and TC are comparing recovery over the same OA surface areas for respective organisms.
