# Supplementary material for: Investigating surface area and recovery efficiency of healthcare-associated pathogens to optimize composite environmental sampling
Source: PLoS One. 2024 Nov 8;19(11):e0310283. doi: 10.1371/journal.pone.0310283 (PMC11548722; doi:10.1371/journal.pone.0310283)
Supplement: S2 Table — A. Conservative sampling areas for each pathogen, standard WT method area, conservative theoretical composite (TC) areas, and ratio of tools required. a Conservative Area of 323 cm2 is the smallest area coupon tested in this study. b Standardized Whole Tool Area of 645 cm2. c Conservative TC area is 1,290 cm2; calculated using the conservative area value of 323 cm2 in Eq 1: (2 × (face + edge)). d Tools Required (WT) represents the ratio of the number of sampling tools required for sampling the optimum area using the WT technique (645 cm2) compared to the number of tools needed using the conservative TC method (1,290 cm2). For example, a ratio of 2:1 means that two tools are needed for the WT method compared to one tool for the conservative TC method. NOTE: This demonstrates the number of tools required to cover the conservative TC area. B. Median CFU recovery of each pathogen: standard WT method as compared to conservative theoretical composite and conservative multiple WT samplings using a conservative area of 1,290 cm2. a Median CFU from standardized whole tool area of 645 cm2 determined in Phase I. b Calculated using Eq 1, (2 × (face + edge)) using median CFU recovered from conservative area. c Calculated CFU (Standard WT x tools needed for same area as conservative TC method in S2A Table). d Recovery comparison represents the percentage of recovery of the conservative TC from the recovery of conservative multiple WT samplings calculated by: ((conservative TC / conservative multiple WT) × 100). * Denotes organisms whereby the TC area is equivalent to the conservative TC area. NOTE: Both conservative multiple WT samplings and conservative TC are comparing recovery over the same surface areas (1290cm2). (DOCX) [file pone.0310283.s002.docx]

# **S2 Table. Summary of conservative TC and WT.**

**A. Conservative sampling areas for each pathogen, standard WT method area, conservative theoretical composite (TC) areas, and ratio of tools required.**

|  | **Conservative Area**^a^ **(cm^2^)** | | **WT Area**^b^ | **Conservative TC Area**^c^ | **Tools Required (WT)**^d^ |
| --- | --- | --- | --- | --- | --- |
|  | **Face** | **Edge** |  |  |  |
| **Gram-negative** |  |  |  |  |  |
| *Acinetobacter baumannii* | 323 | 323 | 645 | 1,290 | **2:1** |
| *Klebsiella pneumoniae* | 323 | 323 | 645 | 1,290 | **2:1** |
| **Gram-positive** |  |  |  |  |  |
| methicillin-resistant *Staphylococcus aureus* | 323 | 323 | 645 | 1,290 | **2:1** |
| *Enterococcus faecalis* | 323 | 323 | 645 | 1,290 | **2:1** |
| **Spore former, Gram-positive** |  |  |  |  |  |
| *Clostridioides difficile* | 323 | 323 | 645 | 1,290 | **2:1** |

^a^ Conservative Area of 323 cm^2^ is the smallest area coupon tested in this study.

^b^ Standardized Whole Tool Area of 645 cm^2^.

^c^ Conservative TC area is 1,290 cm^2^; calculated using the conservative area value of 323 cm^2^ in Equation 1: (2 × (face + edge)).

^d^ Tools Required (WT) represents the ratio of the number of sampling tools required for sampling the optimum area using the WT technique (645 cm²) compared to the number of tools needed using the conservative TC method (1,290 cm²). For example, a ratio of 2:1 means that two tools are needed for the WT method compared to one tool for the conservative TC method.

NOTE: This demonstrates the number of tools required to cover the conservative TC area.

**B. Median CFU recovery of each pathogen: standard WT method as compared to conservative theoretical composite and conservative multiple WT samplings using a conservative area of 1,290 cm^2^.**

|  | **Standard WT**^a^ | **Conservative TC**^b^ | **Conservative Multiple WT Samplings**^c^ | **Recovery Comparison**^d^  (%) |
| --- | --- | --- | --- | --- |
| **Gram-negative** |  |  |  |  |
| *Acinetobacter baumannii* | 4,760 | 9,264 | 9,535 | 97 |
| *Klebsiella pneumoniae* | 813 | 1,389 | 1,629 | 85 |
| **Gram-positive** |  |  |  |  |
| methicillin-resistant *Staphylococcus aureus** | 18,318 | 47,998 | 36,657 | 131 |
| *Enterococcus faecalis** | 27,600 | 79,781 | 55,286 | 144 |
| **Spore former, Gram-positive** |  |  |  |  |
| *Clostridioides difficile* | 10,900 | 14,496 | 21,834 | 66 |

^a^ Median CFU from standardized whole tool area of 645 cm^2^ determined in Phase I.

^b^ Calculated using Equation 1, (2 × (face + edge)) using median CFU recovered from conservative area.

^c^ Calculated CFU (Standard WT x tools needed for same area as conservative TC method in table S2A).

^d^ Recovery comparison represents the percentage of recovery of the conservative TC from the recovery of conservative multiple WT samplings calculated by: ((conservative TC / conservative multiple WT) × 100).

* Denotes organisms whereby the TC area is equivalent to the conservative TC area.

NOTE: Both conservative multiple WT samplings and conservative TC are comparing recovery over the same surface areas (1290cm^2^).
